# Screen time is differentially related to visual selective attention and visual feature processing across middle childhood

**DOI:** 10.21203/rs.3.rs-9204644/v1

**Published:** 2026-04-19

**Authors:** Dilara Keşşafoğlu, Jazlyn Nketia, Dima Amso, Andrew Lynn

**Affiliations:** University of Louisville; Brown University; Columbia University; University of Louisville

**Keywords:** screen time, visual selective attention, visual search, contrast sensitivity, cognitive development

## Abstract

Increased screen time has been linked to attention difficulties in early childhood, raising concerns among parents and professionals. However, it remains unclear how screen use influences the development of visual attention and visual processing across childhood. In this study, we examined associations between daily screen time and performance on visual search and contrast sensitivity tasks in children aged 4–9 years. Screen time was associated with age-related changes in visual search performance, with different patterns observed across task types and visual features. In the conjunction search task, younger children tended to perform better on luminance-based trials at *lower* levels of screen exposure, with age-related changes converging across feature types with more screen time. In contrast, in the feature search task, younger children tended to show better luminance-based performance with *higher* screen exposure. Age-related changes in color-based search tended to remain relatively stable across screen time levels in both tasks. In addition, greater daily screen use was modestly associated with reduced contrast sensitivity across feature channels. Together, these findings suggest that screen time may differentially relate to the development of early visual processing and higher-level visual selective attention, with luminance processing appearing particularly sensitive to variation in daily screen exposure.

## Introduction

Children in the modern era are born into households with numerous digital devices readily accessible to them [1]. Unsurprisingly, screen-based media use is increasingly prevalent among preschool- and school-aged children, with an average of more than three hours of daily use [2] [3]. A growing body of research indicates that increased screen time is associated with attention problems in children, such as inattention, hyperactivity, and distractibility, raising significant concerns among parents and professionals [4] [5] [6] [7]. Although these studies provide important insights into the potential impact of screen use on children’s attention, they primarily rely on indirect parent- or teacher-report measures of children’s attentional processes (e.g., the Child Behavior Checklist [8]) rather than direct assessments. One process that merits investigation is visual selective attention, which may be uniquely affected by screen exposure due to its strong reliance on visual input.

The ability to selectively attend to relevant visual information while ignoring irrelevant information is a critical component of learning and memory [9] [10] [11] [12]. Extensive research indicates that visual selective attention undergoes protracted development, from infancy through childhood and into adolescence [13] [14] [15] [16]. It is often measured using conjunction search tasks, in which participants search for a target object defined by the conjunction of two or more visual features (e.g., red square) among a varying number of spatial distractors that each share one visual feature with the target (e.g., green squares and red circles). A small but growing body of research provides conflicting results, whereby children’s visual attention may be challenged by screen use in some cases and supported in others.

Longitudinal studies of touchscreen use in toddlers (18 to 42 months) show that limited daily screen exposure is associated with faster performance on feature-based visual search (e.g., finding a red apple among blue apples), but slower performance on conjunction search tasks requiring target selection and distractor suppression (e.g., finding a red apple among blue apples and slices of red apples [17] [18]. These findings suggest that screen use may enhance exogenously driven visual orienting while reducing the efficiency of endogenous, goal-directed attentional control in early childhood. Complementing these results, intervention studies demonstrate that interactive storytelling improves visual search performance in 4- to 6-year-olds, whereas passive screen viewing does not, and children exposed to screen-based content show neural patterns associated with reduced attentional control [19] [20]. In contrast, screen time during the hour before bedtime does not appear to impact the visual attention of toddlers aged 16 to 30 months [21]. Overall, these findings indicate that screen use may influence behavioral and neural indices of visual attention in childhood, but the nature of this relationship remains unclear.

Emerging research also suggests that visual selective attention development may not follow one uniform developmental trajectory. Instead, its course may depend on visual feature processing (e.g., color, luminance, motion [22] [15]). Visual feature processing refers to the ability to detect and discriminate visual stimuli (e.g., distinguishing red from green), and is widely assessed using contrast sensitivity and feature search tasks [23] [24] [25]. Studies show that developmental changes in performance on these tasks may depend on the target visual feature and individual differences in visual processing ability. For example, 6- to 7-year-olds are slower to detect an orientation-defined target (e.g., an oblique bar among vertical bars) compared to a color-defined target (e.g., a red bar among purple bars), whereas 9- to 10-year-olds detect both targets with similar speed [24]. Notably, age-related changes in visual feature processing contribute to developmental changes in visual selective attention [14] [15]. Yet, how screen use impacts visual feature processing and its relation with visual selective attention across childhood remains unknown.

Research on action video game play provides one example in which screen-based activity has been linked to enhanced visual processing and attention, including faster reaction times [26] [27], improved contrast sensitivity [28] [29], and more efficient endogenous - attention [30] [31] [32]. These effects are thought to arise from video games promoting more flexible and efficient allocation of attentional resources [26] [33]. However, because children use screens for a wide range of activities beyond video games, it is unclear whether everyday screen exposure has similar effects on foundational visual attention skills.

In the present study, we investigated the relation between daily screen time and visual selective attention in children. We examined a subset of previously reported data, which included a battery of visual search and contrast sensitivity tasks, to assess the associations between screen time, visual selective attention, and visual processing [15]. Performance on such tasks is sensitive to attention development and has been widely used in cognitive developmental research [34] [35]. By analyzing visual search performance as a function of screen time, we aim to characterize how screen exposure duration relates to children’s developing ability to direct visual attention efficiently. This study contributes to the growing literature on screen use and cognitive development by (1) focusing specifically on visual selective attention rather than parent- or teacher-reported attention problems; (2) examining links between screen time and visual processing; and (3) exploring how screen time moderates the age-related changes in visual attention performance. Clarifying these associations may inform educational practices and media-use guidelines during a critical period for attentional development.

## Methods

### Participants

The current sample included a subset of seventy-two 4- to 9-year-old children (*M* = 6.58 years, *SD* = 1.46) and their caregivers drawn from a previous study (*N* = 103) [15]. For the present analyses, we only analyzed data from children who completed at least one computer task and whose caregiver completed a screen use questionnaire (see Table 1 for demographic characteristics). Participants were recruited through advertisements and a database of previous participants.

Children were screened for neurodevelopmental disorders (e.g., Autism, ADHD), learning disabilities (e.g., dyslexia), neurological disorders or injuries (e.g., seizure disorders), and color blindness (using the Ishihara tests for color deficiency). This study and its consent procedures were conducted in accordance with the Declaration of Helsinki and approved by the Institutional Review Board of Brown University. Methods employed in this study were in accordance with relevant guidelines and regulations. Written informed consent was obtained from all parents or legal guardians of the participating children, and verbal assent was obtained from the children prior to participation. Families received $15 for participation.

### Procedure

Children provided verbal assent, and their caregivers provided written informed consent. Children completed a series of computerized tasks in a fixed order on a desktop computer using PsychToolbox and MATLAB software while their caregivers filled out a demographic questionnaire (e.g., child’s age, sex, race, and household income) and a screen use questionnaire. To track progress and maintain motivation, children received a sticker after completing each task, which they placed on a “sticker chart.”

## Materials

### Screen Use Questionnaire

Parents reported their child’s average daily screen time by selecting one of six categories ranging from *less than 1 hour to more than 5 hours*. Midpoints of each category were used to generate numerical estimates (e.g., 1.5 hours for the *1–2 hours* category). Parents also indicated the devices their child could access (at home and outside the home), the purposes of use (e.g., video viewing for entertainment or education), and rated their child’s technology expertise on a 5-point scale (1 = *Needs considerable assistance*; 2 = *Novice*; 3 = *Intermediate*; 4 = *Advanced*; 5 = *Expert/Completely independent*).

### Computer Tasks

Children first completed a contrast sensitivity task, followed by a feature search task and a conjunction visual search task. For extended details of tasks, see [15].

#### Contrast sensitivity task.

Children completed an orientation discrimination task with contrast-modulated Gabor patches that varied in luminance or color (red–green, blue–yellow). On each of 64 trials, a centrally presented Gabor patch appeared either vertically or horizontally, and children indicated its orientation by pressing one of two cartoon-labeled response buttons. Stimulus contrast was adapted using the QUEST+ algorithm, which estimated threshold, slope, and attentional lapse rate (contrast range = −40 to 0 dB; slope = 2–5; guess rate = 0.5; lapse rate = 0–0.04). Contrast thresholds (dB) were converted to Michelson contrast, and log contrast sensitivity was calculated as the inverse of threshold.

#### Visual search tasks.

Stimuli were red, green, black, and white circles (~ 0.5° in diameter) shown at 12 locations around a central cartoon fish. Circles were static or oscillated (~ 0.5° at ~ 1°/s), depending on the motion and/or search condition. Red/green values were luminance-matched; black/white were chromaticity-matched. Luminance contrast was equated across feature conditions. Targets appeared on 50% of trials. Visual Feature (color vs. luminance) and Set Size (1–11, in increments of 2) were manipulated. Trial timing was controlled, and a central image re-centered attention after each trial.

#### Feature search.

Children searched for a color- or luminance-defined target (red or black) among distractors within 2 seconds. Motion was manipulated (static vs. moving) in blocks. Within moving trials, motion was either homogeneous (all items moved in the same direction) or heterogeneous (half vertical, half horizontal). Motion was irrelevant to the task. Visual Feature and Motion conditions were presented in counterbalanced blocks (96 and 48 trials, respectively).

#### Conjunction search.

Children searched for a vertically moving color- or luminance-defined target among vertically and horizontally moving distractors within 3 seconds. Motion was relevant for identifying the target. Each Visual Feature condition (color-motion, luminance-motion) was presented in four 24-trial blocks. Adjacent distractor types (e.g., vertically moving green circle, horizontally moving red circle) were counterbalanced.

#### Dependent Variable.

Performance was computed using *P* = RT(1 + 2ER), where RT was reaction time, and ER was error rate [36]. Only performance on Set Size 3–11 trials was examined, as these trials included spatial distractors.

## Results

We first characterized children’s screen environment, then examined the relation between screen use and children’s performance on the contrast sensitivity, feature search, and conjunction search tasks. We indicated whenever univariate (1.5*IQR) or multivariate outliers (> 1.5IQR, Mahalanobis distance) were detected for each task separately.

### Screen Use

On average, parents reported that children engaged in approximately one and a half hours of daily screen use (see Table 2), had access to an average of five devices at home and two devices outside the home, and that children could use multiple devices efficiently without help. Most parents indicated that mobile devices (e.g., smartphones and tablets) were accessible both at home and outside the home (see Table 3). Parents also reported that their child used technology to play games and watch videos for both entertainment and educational purposes, but were less likely to use technology to search the internet (see Table 4).

### Contrast Sensitivity

After removing twelve univariate outliers and one child who did not complete the contrast sensitivity task, the final sample included 59 children. Log contrast sensitivity values were analyzed using a linear mixed-effects model with Visual Feature (luminance, color-r/g, color-b/y) as a within-subject factor, Age and Screen Time as continuous predictors, and Participant as a random intercept. Previously reported findings were replicated in this subsample [15].

**Figure 1:**
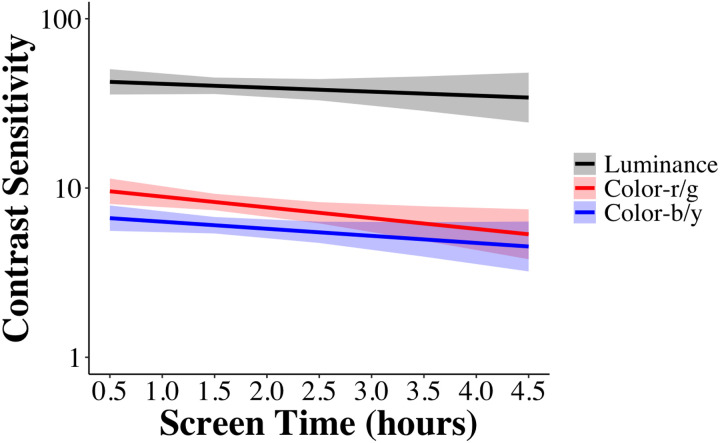
Predicted contrast sensitivity scores across daily screen time, with 95% confidence intervals (shaded). The model indicates a modest but consistent decline in contrast sensitivity with increasing screen exposure.

The analysis revealed a main effect of Screen Time, *F*(1, 55) = 5.12, *p* = .028, *η*_*p*_^*2*^ = .02, but no interactions. These results show that more daily screen use is associated with reduced overall contrast sensitivity, regardless of feature channel (Fig. 1).

### Feature Search

After removing four univariate outliers and two multivariate outliers and one child who did not complete the feature search task, the final sample included 65 children. Log-transformed P values were analyzed using a linear mixed-effects model with Visual Feature (luminance, color) and Motion (static, irrelevant motion) as within-subject fixed factors, Set Size (3, 5, 7, 9, 11), Age, and Screen Time as continuous predictors, and Participant as a random intercept. Previously reported findings were replicated in this subsample [15].

**Figure 2:**
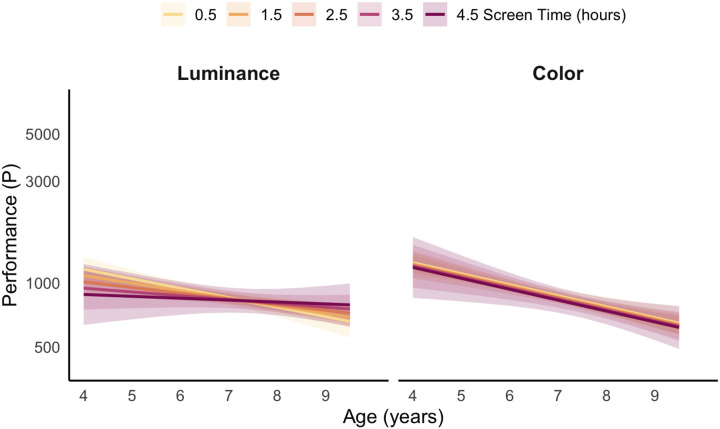
Predicted performance (P) across age for luminance and color feature conditions in the feature search task, with lines representing different levels of daily screen time. Shaded ribbons indicate 95% confidence intervals.

A significant three-way interaction was observed: Visual Feature x Age x Screen Time, *F*(1, 1207) = 5.52, *p* = .019, *η*_*p*_^*2*^ = .005. To follow up on this interaction, simple slopes of age were estimated at five observed levels of daily screen time, separately for luminance and color feature conditions (Fig. 2). Pairwise comparisons of slopes indicated that, across all conditions except at the two highest screen time levels for luminance, performance improved with age; however, the magnitude of this improvement differed as a function of both screen exposure and feature type.

At the lowest screen time level (0.5 h/day), both luminance and color searches showed significant age-related improvement (Luminance: *b* = −0.102, *p* = .0001; Color: *b* = −0.105, *p* < .0001), and the slopes did not differ between features (Δ*b* = 0.003, SE = 0.012, *t*(1207) = 0.24, *p* = .811). At moderate levels of screen time, age slopes remained significant for both feature conditions (1.5 h/day: Luminance: *b* = −0.086, *p* < .0001; Color: *b* = −0.106, *p* < .0001; 2.5 h/day: Luminance: *b* = −0.069, *p* = .0004; Color: *b* = −0.106, *p* < .0001). At these levels, the luminance slope differed significantly from the color slope (1.5 h/day: Δ*b* = 0.020, SE = 0.008, *t*(1207) = 2.57, *p* = .010; 2.5 h/day: Δ*b* = 0.037, SE = 0.009, *t*(1207) = 4.00, *p* = .0001).

At higher levels of screen time, age slopes remained significant for color (3.5 h/day: *b* = −0.106, *p* = .0007; 4.5 h/day: *b* = −0.107, *p* = .016), but not for luminance (3.5 h/day: *b* = −0.053, *p* = .081; 4.5 h/day: *b* = −0.036, *p* = .401). Nevertheless, feature slopes differed significantly at both high screen time levels (3.5 h/day: Δ*b* = 0.053, SE = 0.014, *t*(1207) = 3.67, *p* = .0002; 4.5 h/day: Δ*b* = 0.070, SE = 0.021, *t*(1207) = 3.34, *p* = .0009).

These findings suggest that color-based search shows robust developmental improvement that is largely unaffected by daily screen exposure. In contrast, luminance-based search appears more sensitive to screen exposure, with age-related improvements diminishing as screen time increases. Specifically, as screen time increases, younger children show relatively better performance in luminance-based search, whereas older children show weaker age-related gains. This divergence in developmental trajectories across screen time levels and feature conditions likely underlies the observed three-way interaction.

### Conjunction Search

After removing three univariate outliers and one child who did not complete the conjunction search task, the final sample included 68 children. Log-transformed *P* scores were analyzed using a linear mixed-effects model with Visual Feature (luminance-motion, color-motion) as a within-subject fixed factor, Age, and Screen Time as continuous variables, and Participant with a random intercept and a random slope for Set Size (3, 5, 7, 9, 11). Previously reported findings were replicated in this subsample [15].

A significant three-way interaction was observed: Visual Feature x Age x Screen Time, *F*(1, 522.27) = 6.19, *p* < .013, *η*_*p*_^*2*^ = .01. To follow up on this interaction, simple slopes of age were estimated at five observed levels of daily screen time, separately for luminance and color feature conditions (Fig. 3). Pairwise comparisons of slopes indicated that, across all screen time levels and feature conditions, performance improved with age; however, the magnitude of age-related improvement differed between luminance and color primarily at lower levels of screen exposure.

At the lowest screen time level (0.5 h/day), both luminance and color conjunction searches showed significant age-related improvement (Luminance: *b* = −0.077, *p* = .019; Color: *b* = −0.160, *p* < .0001). At this level, the luminance slope differed significantly from the color slope (Δ*b* = 0.083, SE = 0.020, *t*(524) = 4.18, *p* < .0001). At moderate levels of screen time, age slopes remained significant for both feature conditions (1.5 h/day: Luminance: *b* = −0.102, *p* < .0001; Color: *b* = −0.156, *p* < .0001; 2.5 h/day: Luminance: *b* = −0.126, *p* < .0001; Color: *b* = −0.151, *p* < .0001). Slopes differed between features at 1.5 h/day (Δ*b* = 0.054, SE = 0.013, *t*(524) = 4.30, *p* < .0001) but not at 2.5 h/day (Δ*b* = 0.026, SE = 0.014, *t*(524) = 1.85, *p* = .064).

At higher levels of screen time, age-related improvement remained significant for both luminance and color conjunction search (3.5 h/day: Luminance: *b* = −0.150, *p* = .0001; Color: *b* = −0.147, *p* = .0001; 4.5 h/day: Luminance: *b* = −0.174, *p* = .0015; Color: *b* = −0.143, *p* = .009). At these levels, luminance and color slopes did not differ significantly (3.5 h/day: Δ*b* = −0.003, SE = 0.022, *t*(524) = 0.14, *p* = .888; 4.5 h/day: Δ*b* = −0.032, SE = 0.032, *t*(524) = 0.98, *p* = .327).

These findings suggest that developmental improvements in conjunction search are evident across all levels of screen time for both luminance- and color-based conditions. However, the observed three-way interaction reflects differences in age-related changes across feature conditions that are most pronounced at lower levels of screen exposure. Specifically, at lower screen time levels, younger children show relatively better performance in luminance-based conjunction search. As screen time increases, luminance-related performance diminishes, leading to a convergence of luminance and color developmental trajectories at higher levels of screen exposure.

## Discussion

While prior work has linked screen use to broad attentional difficulties in children, most of these studies have relied on parent or teacher reports rather than direct behavioral measurement [5]. The present study used visual search and contrast sensitivity tasks to characterize how screen time is associated with visual selective attention and visual processing in 4- to 9-year-old children. Overall, the findings indicate that screen time is differentially associated with visual attention and visual processing across middle childhood and moderates age-related improvements in these cognitive processes in a feature-specific manner, with luminance-based processing showing greater sensitivity to screen exposure than color-based processing. However, contrast sensitivity is modestly reduced with increased screen time across both feature channels.

First, in the conjunction search task, developmental improvements were robust across all screen time levels for both luminance- and color-based conditions, indicating continued maturation of selective attention throughout the examined age range. However, screen time moderated these age-related changes. At lower levels of screen exposure, age-related improvement was significantly steeper for color than for luminance targets. This pattern replicates the age-related changes observed in the larger sample [15] and aligns with evidence that chromatic processing may develop more slowly than luminance processing across childhood and adolescence [37] [38]. As screen time increased, the difference between luminance and color conditions was reduced. This effect appears to be driven by younger children with lower screen exposure, whose performance was better relative to their peers with higher screen exposure for luminance-based conjunction search only. These luminance-based conjunction search findings are consistent with prior work showing a negative association between screen time and endogenous attention in toddlers [17] [18].

Second, in the feature search task, screen time also moderated the age-related changes of luminance- and color-based search, albeit in the opposite direction. Color-based search performance again tended to be more similar across screen levels. Age-related changes in the luminance-based feature search decreased with increasing screen time, leading to greater differentiation between luminance- and color-based feature search. In contrast to the conjunction search task, these age-related changes in luminance-based search appeared to stem from better performance among younger children with *more* screen exposure. Importantly, at higher levels of screen time, age-related improvement in the luminance condition was no longer statistically significant, suggesting that younger children showed performance comparable to older children as screen exposure increased. These results of luminance-based search are consistent with prior reports of a positive association between screen time and exogenous attention in toddlers [17] [18].

Taken together, these findings suggest that luminance processing is particularly sensitive to variation in daily screen exposure across middle childhood, but that the direction of this sensitivity depends on the attentional demands of the task. Given evidence that luminance and color processing rely on distinct but overlapping visual neural pathways [39] [40], this feature-specificity may indicate differential environmental sensitivity to screen exposure across dorsal (e.g., motion, contrast, luminance) and ventral (e.g., color, objects, faces) visual pathways. In feature search, which relies more heavily on single-feature detection and bottom-up salience, better performance with more screen exposure may reflect facilitation of younger children’s rapid orienting to salient luminance (but not color) cues. In conjunction search, which requires integrating multiple visual features and suppressing competing distractors, better performance with less screen exposure may reflect facilitation of younger children in top-down attentional control to luminance-defined objects in motion (but not color-defined objects in motion). Thus, these contrasting results are consistent with the idea that habitual screen use may bias attention toward stimulus-driven, exogenous orienting at the expense of endogenous control [41] [42] and suggest that middle childhood may be a sensitive period for dorsal visual pathway development.

Finally, greater daily screen use was associated with reduced contrast sensitivity across luminance and color conditions. This pattern contrasts with findings from the action video game literature, which often reports enhanced contrast sensitivity [43] [28], and may reflect differences in the nature of screen activities, which in young children frequently involve passive viewing or educational content rather than highly engaging gameplay. Video game studies may also employ more specialized psychophysical paradigms designed to capture subtle perceptual gains under certain viewing conditions than the contrast sensitivity task used in the present study. As such, it is important for future work to examine the role of typical child-directed screen media relative to other specific screen use instances, such as for video games. Notably, whereas feature and conjunction search performance showed feature-specific and developmentally constrained associations with screen time, contrast sensitivity showed a more uniform relationship with screen exposure. This dissociation suggests that screen use may exert separable influences on early visual processing and higher-level attentional processes, highlighting the importance of examining both perceptual and cognitive mechanisms when evaluating the impact of screen exposure on developing visual systems.

The present study has several limitations that provide opportunities for future research to better understand the relation between screen use and attention development. First, our cross-sectional design limits our interpretations of developmental change within a child and cannot speak to causal or bidirectional effects. Future studies should employ longitudinal and/or intervention designs to clarify directionality. Second, screen time was parent-reported, which may have introduced recall bias or underestimation of actual use. Objective measures such as digital logs could improve precision in future work. Third, we did not differentiate between types of media content (e.g., gaming vs. passive viewing), which likely vary in their cognitive and attentional demands. Because children’s exposure to specific content types may change with age, future studies should examine these differences to inform more targeted recommendations for screen use across development. Finally, incorporating neurophysiological measures (e.g., EEG, fMRI) could help determine whether screen-related behavioral differences reflect underlying changes in cortical plasticity.

## Conclusions

The present study demonstrates that daily screen use is differentially related to visual selective attention and visual processing across middle childhood. Screen time moderated age-related patterns in both conjunction and feature search, with luminance processing showing greater sensitivity to screen exposure. Screen use was also associated with reduced contrast sensitivity, indicating links to early visual processing as well. These findings suggest that screen exposure is neither uniformly beneficial nor uniformly detrimental, but instead may differentially influence specific perceptual and attentional processes. By employing established visual search and contrast sensitivity tasks to obtain performance-based measures of attention and visual processing, this study contributes novel evidence to the growing literature on screen exposure and cognitive development during a critical period of visual and attentional maturation.

## Figures and Tables

**Table 1:** Demographic characteristics of the sample (N = 72)

Characteristics	*n*	*%*
Child sex		
Female	43	59.7%
Male	29	40.3%
Child race		
White	61	84.7%
Black/African American	3	4.2%
Asian or Pacific Islander	2	2.8%
Native American/American Indian	1	1.4%
Multiracial	4	5.6%
Other	1	1.4%
Ethnicity		
Non-Hispanic	64	88.9%
Hispanic	8	11.1%
Maternal Education		
High school	1	1.4%
Some college	4	5.6%
Associate’s degree	6	8.3%
Bachelor’s degree	30	41.7%
Master’s degree	22	30.6%
Professional/Doctoral degree	8	11.1%
Prefer not to respond	1	1.4%
Paternal Education		
High school	14	19.4%
Some college	8	11.1%
Associate’s degree	6	8.3%
Bachelor’s degree	23	31.9%
Master’s degree	9	12.5%
Professional/Doctoral degree	7	9.8%
Prefer not to respond	5	7.0%
Household income		
Below $75.000	17	23.6%
$75.000 – $170.000	30	41.7%
Above $170.000	12	16.7%
Prefer not to respond	13	18.0%

**Table 2:** Descriptive information (N = 72)

Child age (years)	*M*	*SD*	*Mdn*	*Min*	*Max*
	6.58	1.46	6.30	4.05	9.16
Daily screen use (hours)	1.67	0.98	1.50	0.50	4.50
Screens at home	4.93	1.42	5.00	1.00	8.00
Screens outside home	2.06	1.56	2.00	0.00	6.00
Technology skill level	2.90	1.08	3.00	1.00	5.00

**Table 3:** Availability of devices at home and outside the home

Smartphone/Tablet	Access at home (%)	Access outside home (%)
	100	61.1
Assistants	58.4	11.1
TV	95.8	40.3
Gaming system	48.6	8.3
Computer	87.5	57

**Table 4:** Purposes of children’s technology use

Playing games	Entertainment only (%)	Educational only (%)	Both entertainment & educational (%)	None (%)
	1.4	11.1	73.6	13.9
Watching videos	9.7	2.8	81.9	5.6
Searching the internet	1.4	16.7	19.4	62.5

## Data Availability

The datasets analyzed during the current study are available from the corresponding author on reasonable request.
